# The Gut Microbiota and Respiratory Diseases: New Evidence

**DOI:** 10.1155/2020/2340670

**Published:** 2020-07-31

**Authors:** Li Chunxi, Liu Haiyue, Lin Yanxia, Pan Jianbing, Su Jin

**Affiliations:** ^1^Department of Respiratory and Critical Care Medicine, Nanfang Hospital, Southern Medical University, Guangdong Guangzhou 510515, China; ^2^Department of Laboratory Medicine, Zhujiang Hospital, Southern Medical University, Guangdong Guangzhou 510282, China

## Abstract

Human body surfaces, such as the skin, intestines, and respiratory and urogenital tracts, are colonized by a large number of microorganisms, including bacteria, fungi, and viruses, with the gut being the most densely and extensively colonized organ. The microbiome plays an essential role in immune system development and tissue homeostasis. Gut microbiota dysbiosis not only modulates the immune responses of the gastrointestinal (GI) tract but also impacts the immunity of distal organs, such as the lung, further affecting lung health and respiratory diseases. Here, we review the recent evidence of the correlations and underlying mechanisms of the relationship between the gut microbiota and common respiratory diseases, including asthma, chronic obstructive pulmonary disease (COPD), cystic fibrosis (CF), lung cancer, and respiratory infection, and probiotic development as a therapeutic intervention for these diseases.

## 1. Introduction

Chronic respiratory diseases, including asthma and chronic obstructive pulmonary disease (COPD), as well as respiratory virus infection, are often accompanied by gastrointestinal diseases or symptoms [1–3]. Patients with gastrointestinal diseases, such as inflammatory bowel disease (IBD) and gastroesophageal reflux, are prone to develop pulmonary dysfunction and have an increased incidence of respiratory disease [4, 5]. These connections suggest a vital communication between the gut and lung. The human microbiome is believed to contribute to homeostasis and disease and is responsible for the interactions between these two mucosal sites. Changes in microbial composition or/and diversity will not only directly affect the colonized organ itself but also impact distant organs and systems [6]. In particular, gut microbiota dysbiosis is associated with various diseases, such as allergies, autoimmune diseases, diabetes, obesity, and cancer [7]. Recently, an increasing amount of evidence has indicated that the gut microbiota is closely related to respiratory health and disease, playing a crucial role in the development of asthma, COPD, cystic fibrosis (CF), lung cancer, and respiratory infection [8–11]. In this review, we summarize the recent findings involving the relationship and mechanisms underlying the relationship between the gut microbiota and common respiratory diseases, including asthma, COPD, CF, lung cancer, respiratory infection, and other respiratory diseases, and the use of probiotics for improving or treating these diseases.

## 2. The Gut and Airway Microbiome

The human gastrointestinal (GI) tract harbors approximately 10^14^ bacteria consisting up to 1000 different species [12], and the advance of sequencing technology has rendered the gut microbiota the most widely studied microbiome of the human body. However, the study of the airway microbiota is still in its infancy when compared with that of the gut microbiota. At the phylum level, Firmicutes and Bacteroidetes account for more than 90% of the gut microbial community [13]. The microbiota of the upper and lower respiratory tracts are distinct, with more Firmicutes and Actinobacteria in the nostril and more Firmicutes, Proteobacteria, and Bacteroidetes in the oropharynx [14], whereas there are more Bacteroidetes and Firmicutes in the lung [15]. At the genus level, *Bacteroides*, *Faecalibacterium*, and *Bifidobacterium* are enriched in the gut [16], while *Prevotella*, *Veillonella*, and *Streptococcus* are the prominent genera in the lung [15]. Although the gut and respiratory microbiota exhibit compositional differences, the epithelia of both the GI and respiratory tracts develop from a common embryonic structure, the anatomical structures and functions of the two mucosal sites are similar, and early-life microbial colonization of the gut and lung exhibits similarities. Therefore, accumulating evidence has highlighted the relationship and crosstalk between the gut and lung, referred to as the gut-lung axis [10, 17, 18].

**Figure 1:**
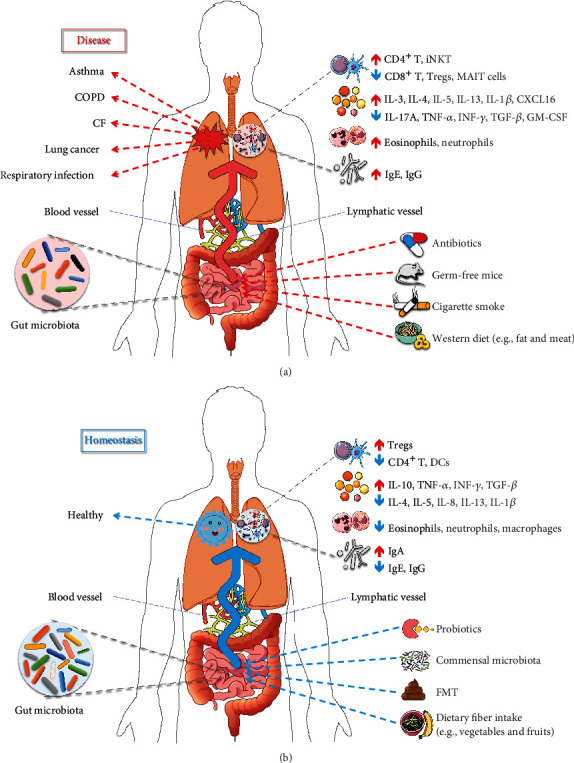
The role of gut microbiota in respiratory disease and homeostasis. The dysbiosis of gut microbiota contributes to respiratory diseases (a), while a healthy gut microbiota plays a protective role in the lung (b). The gut microbiota is influenced by several factors, including antibiotics, probiotics, cigarette smoke, diets, and fecal microbiota transplantation (FMT), and is associated with lung health and disease by regulating the respiratory immunity and inflammation through the blood and lymphatic system. ↑: increase; ↓: decrease.

The gut microbiota is affected by many factors, such as drugs, diet, mode of delivery, and feeding practices, which may play a role in susceptibility to respiratory diseases (Figure 1). For example, early-life acid-suppressive medications and antibiotic use, fast food consumption, caesarian-section delivery, and formula feeding are correlated with an increased risk of asthma, while a higher fiber intake, vaginal delivery, and breastfeeding are negatively correlated with asthma [19–25]. In addition, diet, smoke, and drugs, such as antibiotic and immunosuppressant, can also influence rheumatoid arthritis (RA) and the gut microbiota [26, 27].

## 3. The Gut Microbiota and Respiratory Diseases

### 3.1. Asthma

Asthma is a chronic airway inflammatory disease characterized by reversible airflow restriction and airway hyperresponsiveness. The increasing morbidity and mortality of asthma have made it a serious threat to human health. According to the “hygiene hypothesis,” early-life exposure to specific microbiota constituents is essential for the development and maturity of the immune system, and their absence may increase the susceptibility to asthma and allergic diseases [28]. With the advance of sequencing technologies, an increasing number of studies have revealed a close relationship between the gut microbiota and asthma. The gut microbiota is different between healthy controls (HCs) and asthmatic individuals and is associated with the development of asthma.

Higher microbial diversity is often regarded as beneficial. A recent study showed a connection between low gut microbial diversity in early life and asthma in childhood [29]. Breastfeeding may protect against asthma and allergic disease in children, and gut bacterial diversity was lower in formula-fed infants than in breastfed infants [30]. In addition to microbial diversity, specific gut bacteria have also been found to be closely related to asthma. For instance, *Clostridium* and *Eggerthella lenta* were more abundant in the gut of asthma patients than in that of HCs [31]. Furthermore, the decrease in *Bifidobacterium*, *Akkermansia*, and *Faecalibacterium* abundances and the increase in *Candida* and *Rhodotorula* abundances increased a child's risk of developing allergies and asthma [32], and intestinal colonization by *Clostridium difficile* at 1 month of age was associated with asthma at 6-7 years of age [33]. Therefore, we speculate that the well-balanced commensal microbiota in the GI tract may be beneficial to host health and that reduced microbial diversity may be a marker for underlying pathologic conditions.

Noting the altered gut microbiota in asthma patients, researchers have attempted to modulate the pulmonary immune response as well as prevent and treat asthma through improving the gut microbiota. Arrieta et al. revealed a significant reduction in *Lachnospira*, *Veillonella*, *Faecalibacterium*, and *Rothia* abundances in the gut of asthmatic infants. Furthermore, inoculations of these bacteria in germ-free (GF) mice ameliorated airway inflammation and prevented asthma development [34]. Similar results were obtained in both murine and human studies in which oral administration of *Lactobacillus rhamnosus*, *Lactobacillus casei*, and *Bifidobacterium breve* potentially prevented and treated allergies and asthma [35–37]. Another study indicated that probiotic intervention for pregnant women and their infants who were at a high risk of allergy could protect caesarian-delivered children from allergic disease [38]. Additionally, a double-blind, randomized, placebo-controlled trial of 160 asthmatic children suggested that *Lactobacillus* can reduce asthma severity and improve asthma control [39].

However, other studies have drawn opposite conclusions, finding that probiotics do not have significant benefits in asthmatic children [40]. In these randomized controlled trials (RCTs), probiotic supplementation (such as with *Lactobacillus* and *Bifidobacterium*) for 7 weeks to 6 months was found to have no preventive or therapeutic effects on children with a high risk of asthma or on asthmatic patients. No significant differences in the outcome measures were observed between the probiotic and placebo groups, including the incidence of asthma, clinical outcomes (asthma-related events, quality of life, respiratory tract infections, antibiotic use, and asthma exacerbations), and pulmonary function (fraction of exhaled nitric oxide (FeNO) and forced expiratory volume in 1 s (FEV1)) [41–46]. Although probiotics had no significantly beneficial effects on asthma, the possibility of preventing and treating asthma cannot be denied. In addition, fecal microbiota transplantation (FMT) is another way to improve the gut microbiota, but its clinical application is currently limited in asthma. Additional studies are required to confirm the clinical stability and safety of both probiotic supplementation and FMT. In summary, the gut microbiota is closely associated with asthma, and its imbalance is related to an increased risk and severity of asthma, suggesting that appropriate gut microbiota intervention may be a feasible way to prevent and treat asthma.

Probiotics are also used in patients with autoimmune diseases, such as RA, which have been shown to be associated with the gut microbiota [47]. An early study reported that *Lactobacillus salivarius*, *Lactobacillus iners*, and *Lactobacillus ruminis* were increased in the gut of untreated RA patients, suggesting that a relationship potentially exists between the *Lactobacillus* community and the development of RA [48]. RCTs have demonstrated beneficial the effects of *Lactobacillus acidophilus*, *Lactobacillus casei*, or *Bifidobacterium bifidum* in RA patients [49, 50]. In contrast, Pineda et al. found that *Lactobacillus rhamnosus* and *Lactobacillus reuteri* did not clinically improve RA [51]. Probiotics have been shown to regulate immune system function and affect inflammation in a strain-specific manner. *Lactobacilli* are probiotic bacteria; however, different *Lactobacillus* species have different effects on RA, and some *Lactobacillus* species may cause arthritis [52]. For example, *Lactobacillus casei* plays an important role in inducing arthritis, while *Lactobacillus fermentum* does not induce arthritis [53]. Therefore, regarding probiotic supplementation, it is highly important to choose a bacterial strain and dosage that are safe and beneficial.

### 3.2. COPD

COPD, a common chronic, preventable, and treatable respiratory disease, is characterized by persistent airflow limitation and increased airway inflammation. Worldwide, COPD has been a major public health problem because of its high prevalence, morbidity, and mortality. Although much evidence has shown a coexistence of COPD and chronic gastrointestinal diseases such as IBD, few studies have reported the gut microbiota in COPD patients. Smoking is the principal cause of developing COPD and is associated with the microbial community and immune response of the GI tract [54]. The gut microbiota changes along with different cigarette smoking statuses [55]; Biedermann et al. found an altered gut microbiota in healthy smokers compared with that in nonsmokers, and they further observed an increase in Actinobacteria and Firmicutes and a decrease in Bacteroidetes and Proteobacteria abundances after smoking cessation [56]. In a mouse study, Lachnospiraceae sp. was increased in the gut after smoke exposure [54]. Although few studies have identified the direct association between the gut microbiota and COPD, there is evidence that the gut microbiota may play a vital role in COPD induced by cigarette smoke.

Similarly, there have been relatively few studies about probiotics that revealed the connection between the gut microbiota and COPD. For example, intragastric supplementation with *Lactobacillus rhamnosus* and *Bifidobacterium breve* in mice with COPD attenuated airway inflammation and alveolar damage [57]. *In vitro*, these two probiotics showed a similar anti-inflammatory effect on cigarette smoke-induced inflammation in human macrophages [58]. In the future, additional studies conducted on COPD patients are required to investigate and confirm the role of probiotics in COPD and to provide new therapeutic strategies for COPD.

### 3.3. CF

CF, a common autosomal recessive disease that affects mainly the lungs, is primarily driven by cystic fibrosis transmembrane conductance regulator (CFTR) mutation. The GI tract also strongly presents CFTR dysfunction and is among the earliest parts of the body affected in CF patients, suggesting a close link between the gut and lung. In CF patients, the gut microbiota was significantly altered, with reduced bacterial abundance, richness, and diversity and different microbial compositions compared to those in HCs [59–61]. For example, increased abundances of *Staphylococcus*, *Streptococcus*, and *Veillonella dispar* and decreased abundances of Bacteroides, *Bifidobacterium adolescentis*, and *Faecalibacterium prausnitzii* were observed in the gut of CF patients compared with those of HCs [62]. Importantly, the gut microbiota, which was reported to be associated with CFTR variants [63], seems to be essential for the pathophysiology and development of CF. A murine study indicated that the loss of functional CFTR was associated with augmentation of pathogenic bacteria, such as *Mycobacteria* and *Bacteroides fragilis* [64]. Moreover, several cross-sectional studies revealed a certain relationship between the gut microbiota and lung function, disease exacerbation, and severity of CF patients [65–67].

In recent years, numerous RCTs have shown that restoration of the gut microbiota followed by probiotic supplementation is related to improvement of CF, further strengthening the idea that the gut microbiota can influence airway inflammation in CF. *Lactobacillus* administration caused a reduction in bacterial density and an increase in microbial diversity in the gut [68], as well as beneficial effects on exacerbation risk and quality of life in CF patients [69]. However, some inconsistent results were also yielded; for example, Van Biervliet et al. found no significant differences in pulmonary function and disease exacerbations between probiotic and placebo groups [70]. Therefore, according to a meta-analysis, fastidiously designed and adequate RCTs are needed to assess the safety and efficacy of probiotics and to ascertain the specific probiotic strains or dose that can be of significant benefit for CF patients [71].

### 3.4. Lung Cancer

Lung cancer is one of the malignant tumors with the fastest growth of morbidity and mortality and has become the greatest threat to human health. Antibiotics are believed to alter the gut microbiota, and a large demographic study found that exposure to certain antibiotics, such as penicillin, cephalosporins, or macrolides, was associated with an increased risk of lung cancer [72], which suggested a close correlation between the gut microbiota and lung cancer. Using 16S rRNA sequencing, researchers found no significant difference in alpha diversity but a difference in gut microbiota beta diversity between patients with lung cancer and HCs [73, 74]. Moreover, at the phylum and genus levels, lung cancer patients had an increased abundance of *Enterococcus* and a reduced level of the phylum Actinobacteria and genus *Bifidobacterium*, and these microbial communities might be potential biomarkers for lung carcinogenesis [73].

Recently, several studies have indicated that the gut microbiota also contributes to the effect of lung cancer therapeutics. Patients with non-small-cell lung cancer (NSCLC) who responded to antiprogrammed death 1 (PD-1) immunotherapy (responders) harbored a higher gut microbial diversity than those who did not respond (nonresponders), and gut microbial diversity was positively associated with progression-free survival (PFS) [75]. Another study revealed that responders showed increased abundances of *Akkermansia muciniphila*, *Ruminococcus*, *Eubacterium*, and *Alistipes* and decreased abundances of *Bifidobacterium* and *Parabacteroides* in the gut compared with those of nonresponders [76]. In addition, antibiotic use can influence the efficacy of lung cancer therapy. Previous studies found that antibiotics before and during antitumor therapy significantly reduced the clinical benefit (PFS and overall survival) of antitumor drugs in patients with NSCLC [77, 78]. Furthermore, FMT into GF or antibiotic-treated mice can ameliorate antitumor effects and clinical activity of antitumor drugs [79]. Similarly, supplementation with *Enterococcus hirae* and *Barnesiella intestinihominis* can prolong PFS in mice with advanced lung cancer undergoing chemoimmunotherapy [80]. Taken together, the gut microbiota markedly influences the outcome of antitumor therapy for lung cancer, suggesting a potential strategy to improve the clinical outcomes of patients with lung cancer by modulating the gut microbiota. However, a large number of clinical studies are required to confirm the effectiveness and safety of FMT and probiotics.

### 3.5. Respiratory Infection

Respiratory infection is the most common infectious disease and is a leading cause of morbidity and mortality worldwide. The gut commensal microbiota provides essential benefits to pulmonary mucosal immunity and plays protective roles in respiratory infection by distally driving host responses to pneumonia [81]. Depletion or absence of the gut microbiota is believed to influence the host immune response. Schuijt et al. found that microbiota-depleted mice showed increased bacterial dissemination, inflammation, organ damage, and mortality compared with control mice, and FMT reversed the gut microbiota diversity and enhanced the host defense against pneumonia [82]. In addition, the gut microbiota differed between patients with respiratory infection and HCs. It was reported that certain gut microbiota, such as Enterococcaceae, was associated with community-acquired pneumonia (CAP) [83], and respiratory syncytial virus and influenza virus infection resulted in a dysbiotic gut microbiota in mice [84]. Many studies have suggested that oral administration of probiotics can not only protect against bacterial pneumonia [85] but also contribute to accelerated recovery from respiratory viral infection [86, 87], further emphasizing the crucial role of the gut microbiota in respiratory infection.

Tuberculosis (TB) typically affects the lungs (pulmonary TB), causing approximately 10 million cases and over 1 million deaths per year worldwide, with the heaviest toll in low- and middle-income countries [88]. Likewise, the gut commensal microbiota can protect against early lung colonization by *Mycobacterium tuberculosis* (*Mtb*) [89]. Disruption of the gut microbiota with antibiotics increased the burden and dissemination of *Mtb*, and FMT reconstituted the gut microbiota and restored TB containment by reducing the *Mtb* burden [90]. The gut microbiota was significantly different between patients with TB and HCs. At the phylum level, Actinobacteria and Proteobacteria, which contain many pathogenic species, were enriched in the gut of TB patients, while Bacteroidetes, which contains a variety of beneficial commensal microbiota species, was decreased in TB patients compared to those in HCs [91]. At the genus level, several butyrate and propionate-producing bacteria, such as *Faecalibacterium*, *Roseburia*, *Eubacterium*, and *Phascolarctobacterium*, were more abundant in TB patients than in HCs [92]. Similar to antibiotic and antitumor therapy, anti-TB treatment also has dramatic effects on the gut microbiota. Patients who underwent standard HRZE (isoniazid, rifampicin, pyrazinamide, and ethambutol) therapy exhibited a perturbed gut microbiota, with a depletion of *Ruminococcus*, *Eubacterium*, *Lactobacillus*, and *Bacteroides* and an increase in *Erysipelatoclostridium* and *Prevotella* abundances [93], and HRZ(E)-induced dysbiosis was long lasting in both mice and humans [94]. Furthermore, studies on probiotics suggested that supplementation with Lactobacillus can restore anti-*Mtb* immunity in the lungs [95]. Taken together, these findings indicate that the gut microbiota may contribute to the pathophysiology of TB.

### 3.6. Other Respiratory Diseases

In addition to the above common respiratory diseases, other respiratory disorders, such as ILD, acute respiratory distress syndrome (ARDS), acute lung injury (ALI), and ventilator-associated pneumonia (VAP), also show a certain correlation with the gut microbiota. For example, ILD is characterized by progressive fibrosis and respiratory failure, and changes in the gut microbiota have been reported in patients with silicosis and pulmonary fibrosis [96]. ARDS/ALI is the most common form of organ failure in critically ill patients, and VAP, which is among the most common infections in mechanically ventilated patients, has a high mortality rate [97, 98]. Previous studies revealed that gut-associated bacteria and pathogens were enriched in the bronchoalveolar lavage fluid (BALF) of patients with ARDS, which suggested gut-lung translocation [99, 100]. Additionally, the GI microbiota contributes to the development of ALI in mice [101], and FMT can significantly reduce ALI inflammation [102]. RCTs of ventilated patients suggested that patients treated with probiotics had a decreased incidence of microbiologically confirmed VAP, as well as reduced durations of intensive care unit [103] and hospital stays [104]. A meta-analysis also found an association between probiotic supplementation and reduced VAP incidence, suggesting a clinical benefit of probiotics for ventilated patients [105].

## 4. The Complex Interactions between the Gut and Lungs

Cigarette smoking, which is a risk factor for many diseases, can not only change the lung microbiota but also affect the gut microbiota [55, 106]. Lung mucosal exposure to cigarette smoke may be involved in the development of autoantibodies associated with RA, such as peptidylarginine deiminase (PAD) 2 [107], suggesting that the lungs could be a site of autoimmunity generation in RA. Scher et al. found that the lung microbiota in RA patients was similar to that in sarcoidosis patients, characterized by reduced alpha diversity and decreased abundance of *Actinomyces* and *Burkholderia* [108]. The notion that the gut microbiota influences the local and systemic immune systems is not novel, and the role of the gut microbiota in autoimmune diseases, including RA, is currently well characterized [47]. Since the lungs and gut are both mucosal sites that are exposed to environmental factors, it is possible that both organs share microbiota and that the microbiota can induce local and systemic immunity/inflammation in both organs. This evidence suggests that the gut/lung microbiota may potentially drive the initiation of autoimmune diseases.

Previous studies have found an altered function and structure in intestinal mucosa in asthma patients and increased intestinal permeability in COPD patients [109, 110], further supporting the hypothesis that a link exists between the gut and lungs. Moreover, a growing number of studies have suggested that an immunological relationship exists between the gut and lungs [111, 112]. The gut microbiota can shape local intestinal and systemic immunity and the lung mucosa, thereby affecting respiratory diseases. Taken together, the complex interaction between the gut and lungs is likely to be mediated by locally resident microbiota.

## 5. Possible Mechanisms of the Gut Microbiota in Respiratory Diseases

The gut commensal microbiota contributes to influencing and maintaining body homeostasis by regulating the immune response of both the GI system and distal organs. The possible mechanisms include the regulation of extraintestinal T cell populations, development of oral immune tolerance through regulatory T cells (Tregs), production of short-chain fatty acids (SCFAs), and regulation of systemic inflammation [113]. The immune cells and cytokines induced by the gut microbiota and its metabolites, such as SCFAs, can enter systemic circulation through the blood and lymphatic system, which regulate the immune and inflammatory responses in the lung and further influence respiratory health and disease (Figure 1). For example, exaggerated allergic airway inflammation in GF mice was correlated with increased T helper 2 (Th2) cytokine (IL-4 and IL-5) and IgE levels in the lung [114]. The commensal gut microbiota can enhance host defense against bacterial pneumonia by increasing IL-17A levels and upregulating pulmonary granulocyte-macrophage colony-stimulating factor (GM-CSF) signaling [115]. In antibiotic-treated mice, a greatly increased mortality due to respiratory viral infection was related to a decreased abundance of Tregs in the respiratory and GI tracts [116], and increased pulmonary colonization by *Mtb* was associated with a significantly reduced accumulation of mucosal-associated invariant T (MAIT) cells in the lungs [89].  Table 1 summarizes our current understanding of the possible mechanisms of the gut microbiota acting on common respiratory diseases.

Currently, the mechanisms of probiotic regulation of lung health and disease have become a research hotspot since there is increasing evidence that probiotics have protective and therapeutic effects on respiratory diseases by optimizing microbial balance in the GI tract (Table 1). Oral administration of probiotics contributes to regulating respiratory immune responses through numerous signaling pathways. For example, *Bifidobacterium bifidum* can stimulate the Th1/Th2 balance and upregulate IFN-*γ*, IL-4, and IL-12 secretion in the spleen [139]; *Escherichia coli* can reduce respiratory inflammatory cell recruitment as well as Th2 and Th17 responses [140]; *Enterococcus faecalis* suppresses Th17 cell development in the lung, spleen, and gut [141]; and *Lactobacillus plantarum* can reduce the numbers of lung innate immune cells (macrophages and neutrophils) and levels of cytokines (IL-6 and TNF-*α*) in the BALF and induce an immunosuppressive Treg response in the lungs [142]. Despite these effects, the precise mechanisms underlying probiotic effects on the lung and many aspects of the probiotic regulation of immune responses remain largely unknown.

## 6. Conclusions

Increasing evidence suggests an important and complex crosstalk between the gut and lung, as well as between the gut microbiota and host immunity. Gut microbial dysbiosis is believed to be associated with the etiology or/and development of common respiratory diseases, such as asthma, COPD, CF, lung cancer, and respiratory infection. To date, the understanding of the mechanism involving the gut-lung axis is still in its infancy and remains to be further elucidated. Future research into modification and improvement of the gut microbiota and into the balance of gut and lung immunity through diet, probiotics, and FMT is necessary to improve our understanding of the role of gut microbiota in the lung and to provide effective and new therapeutic strategies for respiratory diseases.

## Figures and Tables

**Table 1:** The possible mechanisms underlying the effects of the gut microbiota on respiratory diseases.

Respiratory diseases	Alterations in the gut microbiota	Possible mechanisms	References
Asthma	Gut microbiota disrupted by antibiotics	Exacerbate Th2 responses by increasing the infiltration of inflammatory cells and the production of inflammatory cytokines (IL-4 and IL-13).	[117, 118]
		Reduce Treg abundance in the lung.	[119]
		Exaggerate Th1/Th17 adaptive immune responses in the lung.	[120]
	GF mice	Elevate the total number of eosinophils, number of CD4^+^ T cells, and level of Th2 cytokines and alter the number and phenotype of conventional DCs in the airways.	[114]
		Increase CXCL16 expression and accumulate iNKT cells in the gut and lungs.	[121]
	Probiotics	Reverse the Th1/Th2 imbalance: increase the levels of the anti-inflammatory cytokine IL-10 while reduce the levels of proinflammatory cytokines such as IL-4, IL-5, and IL-13.	[122–125]
		Increase PPAR*γ* expression of DCs in the lung.	[126]
		Increase lung CD4^+^ T cell and CD4^+^Foxp3^+^ Treg abundance while decrease activated CD11b^+^ DC abundance.	[37]
		Decrease MMP9 expression in the BALF and serum and inhibit inflammatory cell infiltration into the lung.	[36]

COPD	Cigarette smoke	Alter mucin gene expression and cytokine production in the gut; increase Muc2, Muc3, and Muc4 expression; and increase CXCL2 and IL-6 expression while decrease IFN-*γ* and TGF-*β* expression.	[54]
		Inhibit the NK-*κ*B pathway by reducing p65 phosphorylation and I*κ*B*α* in the gut.	[127]
	Probiotics	Suppress macrophage inflammation by inducing the expression of IL-1*β*, IL-6, IL-10, IL-23, TNF-*α*, CXCL-8, and HMGB1.	[58]
		Increase NK cell activity and the number of CD16^+^ cells.	[128]

CF	Probiotics	Reduce IL-8 production by intestinal cells.	[129]
		Reduce the level of the gut inflammatory marker calprotectin.	[68]
	Antibiotic treatment	Augment the proportions of Th17, CD8^+^ IL-17^+^, and CD8^+^ IFN*γ*^+^ lymphocytes and IL-17-producing *γδ* T cells.	[130]

Lung cancer	Gut microbiota disrupted by antibiotics	Upregulate the expression of VEGFA and downregulate the expression of BAX and CDKN1B while reduce IFN-*γ*, GZMB, and PRF1 produced by CD8^+^ T cells.	[131]
		Suppress CTX-induced Th17 responses and reduce the abundance of tumor-infiltrating CD3^+^ T cells and Th1 cells.	[132]
	FMT	Accumulate CCR9^+^CXCR3^+^CD4^+^ T cells into the tumor microenvironment.	[79]
	Probiotics	Upregulate the mRNA levels of IFN-*γ*, GZMB, and PRF1.	[131]
		Boost CTX-induced anticancer Th1 and Tc1 responses and promote the infiltration of IFN-*γ*^+^*γδ*T cells into cancer lesions.	[80]

Respiratory infection	Commensal gut microbiota	SFB promotes pulmonary Th17 immunity as demonstrated by increased IL-22 and IL-22^+^ TCR*β*^+^ cell levels.	[133]
		Protect against *Mtb* infection by improving the activity of MAIT cells in the lungs.	[89]
		Regulate virus-specific CD4 and CD8 T cell and antibody responses.	[134]
		Contribute to the accumulation of IL-22-producing ILC3s in newborn lung.	[81]
		Induce NF-*κ*B activation in the lung through TLR4.	[135, 136]
	Gut microbiota disrupted by antibiotics	Reduce pulmonary GM-CSF production through IL-17A signaling.	[115]
		Reduce MAIT cell and IL-17A levels.	[89]
		Reduce mincle expression on lung DCs.	[95]
		Decrease bacterial killing activity of alveolar macrophages while increase the levels of proinflammatory cytokines such as IL-6 and IL-1*β* in the lung.	[136]
	GF mice	Decrease proinflammatory cytokine (TNF-*α* and CXCL1) levels and neutrophil influx while produce large amounts of IL-10 in the lungs.	[137]
	FMT	Normalize the pulmonary TNF-*α* and IL-10 levels.	[82]
	Probiotics	Activate the TLR-signaling pathway through the protein Mal.	[85]
		Enhance the mRNA expression of IFN-*γ*, IL-12a, IL-2rb, IL-12rb1, PRF1, Klrk1, CD247, and TNF-*α* in the lung.	[138]

ALI	FMT	Reduce TNF-*α*, IL-1*β*, and IL-6 levels by downregulating the TGF-*β*1/Smads/ERK signaling pathway.	[102]

ALI: acute lung injury; BAX: Bcl-2 associated X; CDKN1B: cyclin-dependent kinase inhibitor 1B; CTX: cyclophosphamide; CXCL: C-X-C motif chemokine ligand; DCs: dendritic cells; ERK: extracellular signal-regulated kinase; FMT: fecal microbiota transplantation; GF mice: germ-free mice; GM-CSF: granulocyte-macrophage colony-stimulating factor; GZMB: granzyme B; HMGB1: high-mobility group box 1; IFN-*γ*: interferon-gamma; I*κ*B*α*: inhibitor of NK-*κ*B *α*; Klrk1: killer cell lectin-like receptor subfamily K, member 1; MAIT cells: mucosal-associated invariant T cells; Mal: MyD88 (myeloid differentiation primary response protein) adaptor protein; MMP9: matrix metalloproteinase 9; Muc: mucin; NF-*κ*B: nuclear factor kappa-B; PPAR*γ*: peroxisome proliferator-activated receptor gamma; PRF1: perforin; SFB: segmented filamentous bacteria; TLR: toll-like receptor; TNF-*α*: tumor necrosis factor alpha; Tregs: regulatory T cells; VEGFA: vascular endothelial growth factor A.
